# PP18 Effectiveness And Safety Of Long-Acting Agents-Based Dual Therapy In HIV-1/AIDS Patients: A Network Meta-Analysis Of Observational Studies

**DOI:** 10.1017/S0266462325102389

**Published:** 2025-12-29

**Authors:** Daniela Gorski, Bruno Gonçalves Ferreira, Helena Hiemisch Lobo Borba, Fernanda Stumpf Tonin, Roberto Pontarolo

## Abstract

**Introduction:**

Most treatment regimens for people living with HIV (PLHIV) require the intake of multiple daily pills, which can pose challenges to adherence. As an alternative, long-acting (LA) dual regimens have gained attention for their potential to simplify treatment. Given the importance of evaluating the long-term effects of LA regimens, this study aimed to synthesize evidence on their effectiveness and safety.

**Methods:**

A systematic review was performed following the Cochrane Collaboration recommendations and the Meta-Analysis of Observational Studies in Epidemiology (MOOSE) guidelines, with searches in PubMed, Scopus, and Web of Science (July 2024) (PROSPERO-CRD42024570754). Longitudinal studies evaluating any dual-therapy antiretroviral regimen with LA agents (islatravir, cabotegravir, lenacapavir, rilpivirine, albuvirtide) for PLHIV were included. For each outcome of interest, data were pooled using bivariate meta-analysis (discontinuations due to adverse events) and network meta-analysis (NMA) (virologic failure [VF]), with results presented as risk ratios (RR) with 95 percent credibility intervals (CrIs); heterogeneity was assessed using the I-squared statistic. The surface under the cumulative rating curve analysis (SUCRA) was also conducted for the NMA.

**Results:**

Thirty-two studies (2016 to 2024) evaluating 11 treatments were included. Four cohorts (n=2,570) were assessed in the NMA of VF, where the rilpivirine/dolutegravir (RPV/DTG) regimen was superior to non-LA regimens dolutegravir/lamivudine (RR −1.60, 95% CrI: −4.08, 0.91) and dolutegravir/doravirine (RR −0.42, 95% CrI: −1.68, 0.68), with SUCRA probabilities of 9.6 percent, 31 percent, and 59.5 percent, respectively. The triplet regimens dolutegravir plus tenofovir/emtricitabine and dolutegravir plus abacavir/lamivudine had the highest probabilities of leading to CD4+ count increases at six to 24 months (65.6 to 85.7% and 65.8 to 84.4%, respectively), followed by RPV/DTG (59 to 60%) and dolutegravir/lamivudine (46 to 53%). However, RPV/DTG regimens were considered safer than dolutegravir/lamivudine (RR 0.53, 95% CrI: 0.32, 0.89) regarding discontinuation due to adverse events.

**Conclusions:**

This updated synthetized evidence suggested that the RPV/DTG regimen is a promising alternative for simplifying the treatment of PLHIV, demonstrating comparable effectiveness to the dolutegravir/lamivudine and dolutegravir/doravirine regimens, while offering better tolerability. However, considerations of drug accessibility and cost must be carefully weighed in the decision-making process in different settings.

